# Detection of Pathogens in Blood for Diagnosis of Sepsis and Beyond

**DOI:** 10.1016/j.ebiom.2016.06.030

**Published:** 2016-06-23

**Authors:** I. Martin Sheldon

**Affiliations:** Institute of Life Science, Swansea University Medical School, Swansea, United Kingdom

Determining whether a patient has a microbial infection is a common clinical challenge. Sepsis is a case in point where clinical signs may be confused with causes other than infection, such as trauma. In this issue of *EBioMedicine*, Cartwright et al. describe a rapid blood test to discriminate between patients with microbial infections and those with sterile trauma (Cartwright et al., 2016). This is a ground-breaking and much-needed development.

Sepsis is the most common cause of death in hospitalized patients, with an estimated 200,000 deaths annually in the USA (Deutschman and Tracey, 2014). However, sepsis is an imprecise clinical syndrome, with a variable clinical presentation (Angus and van der Poll, 2013). Diagnosis is usually based on suspicion of infection, combined with signs of organ dysfunction (Cohen et al., 2015). Early diagnosis of sepsis and administration of antibiotics is vital because progression to severe sepsis or septic shock has serious consequences (Angus and van der Poll, 2013). Unfortunately, differentiating between sepsis and other inflammatory conditions is often challenging in seriously ill patients. Detecting bacterial infections in blood is a key step in the diagnosis of sepsis, and initiating treatment with antimicrobials (Deutschman and Tracey, 2014, Cohen et al., 2015). However, blood cultures are negative in 60 to 70% of patients with severe sepsis (Cohen et al., 2015), and > 80% were negative in the study by Cartwright et al. (2016). In addition, microbiology takes too long to influence first line therapy against pathogenic bacteria. Developments in PCR and mass spectrometry have increased the likelihood of identifying bacteria in blood samples, but often rely on time-consuming pre-analytical processing such as blood culture in order to increase pathogen load. Proxies for infection include increased circulating cytokines and acute phase proteins, such as C-reactive protein; although, their concentrations also increase during physiological events such as parturition, or pathological tissue damage such as burns.

Pathogen-associated molecular patterns (PAMPs) are molecules found in prokaryotes, such as bacteria and fungi, but not in animals (Akira et al., 2006). Innate immunity relies on the binding of PAMPs to receptors on immune cells and to serum proteins, such as mannose-binding lectin (Akira et al., 2006, Ip et al., 2009). Cartwright et al. developed an ELISA using an engineered immunoglobulin domain fused to domains of mannose-binding lectin. This protein binds carbohydrate PAMPs, including lipopolysaccharide and lipoteichoic acid, from a wide range of Gram-negative and Gram-positive bacteria, and mannan from fungi (Kang et al., 2014). The ELISA detected PAMPs from live or dead pathogenic bacteria in whole blood (Cartwright et al., 2016). Animal studies were used to demonstrate the utility of the assay for detecting PAMPs, even when blood cultures were culture-negative, during antimicrobial treatment, or when the initial infection was intraperitoneal. The ELISA was then evaluated in human patients, and had greater specificity than C-reactive protein in differentiating between infection and trauma. In addition, the assay detected PAMPs in 80% of blood samples from emergency department patients with infection-related diseases and suspected sepsis, even though < 20% had positive blood cultures (Cartwright et al., 2016).

The ELISA developed by Cartwright et al. has several benefits over current diagnostics for infections. First, the assay detects pathogen molecules, even in patients treated with antimicrobials, which often yield negative blood-cultures. Furthermore, the ELISA can be completed within an hour using whole blood, rather than > 24 h often necessary for blood culture (Cohen et al., 2015). Although PCR-based assays are useful when living bacteria are present in blood, the present ELISA also detects PAMPs released during tissue infections even when blood cultures do not detect live pathogens. However, adoption of the ELISA will likely be contingent on large trials to test the predictive value in patient populations with suspected sepsis. Conversely, one wonders whether other assays could be developed to aid the diagnosis of non-infectious trauma, perhaps by detecting damage-associated molecular patterns released by dying or damaged cells (Chen and Nunez, 2010).

Beyond sepsis, overprescribing antibiotics and the rise of antimicrobial resistance is of considerable concern. The O'Neill report, which provides recommendations for tackling drug-resistant infections globally, advises developing diagnostics for bacterial infections to guide the use of antimicrobials (O'Neill, 2016). So, the ELISA described by Cartwright et al. is a welcome step forward to help direct therapy toward patients with bacterial infections. The use of antimicrobials in animals is another concern of the O'Neill report (O'Neill, 2016), and a test for bacterial infection might also find utility with veterinarians to justify the use of antibiotics, especially as the ELISA works in animal models (Cartwright et al., 2016). The ELISA might also be engineered to make a point-of-care assay, facilitating rapid patient treatment, and applicable for emergency care in the field or in developing countries. However, as the ELISA is non-specific, it might need to be paired with molecular diagnostic assays that identify species of bacteria, and perhaps their antimicrobial resistance genes, to best inform the selection of antimicrobials.

In summary, the ELISA developed by Cartwright et al. (2016) provides the potential to use whole blood for the rapid detection of infections. Translation of this technology for use in clinical practice will benefit the diagnosis of sepsis. Beyond sepsis diagnosis, the work may herald methods to meet the clinicians' dream of discriminating infection from sterile inflammation.

## Disclosure

The author declared no conflict of interest. The author's work is supported by the Biotechnology and Biological Sciences Research Council (Grant BB/K006592/1), and Life Sciences Research Network Wales, an initiative funded through the Welsh Government's Ser Cymru program (Grant NRNRG4Mar010).
